# Low FNDC5/Irisin expression is associated with aggressive phenotypes in gastric cancer

**DOI:** 10.3389/fphar.2022.981201

**Published:** 2022-10-28

**Authors:** Luyun Xu, Yan Ye, Yuqin Sun, Wenting Zhong, Liangjie Chi, Youyu Lin, Hongxia Liu, ShengZhao Li, Hui Chen, Chengcheng Li, Yuxuan Lin, Qingshui Wang, Fangqin Xue, Yao Lin

**Affiliations:** ^1^ Central Laboratory at the Second Affiliated Hospital of Fujian Traditional Chinese Medical University, Innovation and Transformation Center, Fujian University of Traditional Chinese Medicine, Fuzhou, China; ^2^ College of Life Sciences, Fujian Normal University, Fuzhou, China; ^3^ Department of General Surgery, Zhangzhou Affiliated Hospital of Fujian Medical University, Zhangzhou, Fujian, China; ^4^ Department of Gastrointestinal Surgery, Shengli Clinical Medical College of Fujian Medical University, Fujian Provincial Hospital, Fuzhou, China; ^5^ Fujian Provincial Key Laboratory of Hepatic Drug Research, Fuzhou, China

**Keywords:** gastric cancer, FNDC5, irisin, KLF9, methylation, immune

## Abstract

**Background:** FNDC5 belongs to the family of proteins called fibronectin type III domain-containing which carry out a variety of functions. The expression of FNDC5 is associated with the occurrence and development of tumors. However, the role of FNDC5 in gastric cancer remains relatively unknown.

**Methods:** In the research, the expression of FNDC5 and its value for the prognosis of gastric cancer patients were observed with the TCGA database and GEO datasets of gastric cancer patients. The role of FNDC5 in the regulation of gastric cancer cells proliferation, invasion, and migration was determined. WGCNA and Enrichment analysis was performed on genes co-expressed with FNDC5 to identify potential FNDC5-related signaling pathways. Meanwhile, the LASSO Cox regression analysis based on FNDC5-related genes develops a risk score to predict the survival of gastric cancer patients.

**Results:** The expression of FNDC5 was decreased in gastric cancer tissues compared to normal gastric tissues. However, survival analysis indicated that lower FNDC5 mRNA levels were associated with better overall survival and disease-free survival in gastric cancer patients. Meanwhile, a significant negative correlation was found between FNDC5 and the abundance of CD4^+^ memory T cells in gastric cancer. *In vitro* overexpression of FNDC5 inhibits the migration and invasion of gastric cancer cells, without affecting proliferation. Finally, A two-gene risk score module based on FNDC5 co-expressed gene was built to predict the overall clinical ending of patients.

**Conclusion:** FNDC5 is low expressed in gastric cancer and low FNDC5 predicts a better prognosis. The better prognosis of low FNDC5 expression may be attributed to the increased number of CD4^+^ memory activated T-cell infiltration in tumors, but the exact mechanism of the effect needs to be further explored. Overexpressing FNDC5 inhibits the invasion and migration of gastric cancer but does not affect proliferation. At last, we constructed a clinical risk score model composed of two FNDC5-related genes, and this model may help lay the foundation for further in-depth research on the individualized treatment of gastric cancer patients.

## Introduction

Gastric cancer (GC) is a malignant tumor of the digestive tract with the second-highest mortality rate and the incidence is increasing annually (Maak et al., 2021). Multifactorial etiology of gastric cancer means that both environmental and genetic factors contribute to the development of the disease. Studies have shown that inflammatory gastrointestinal disease, dietary habits, smoking and alcohol consumption, and genetics are risk factors for GC (Rabiee et al., 2020). Although the main interventions for gastric cancer are surgery, chemotherapy, and drugs, their prognosis is poor due to the lack of specific symptoms and diagnostic markers for early GC (Joshi and Badgwell, 2021), Therefore, further research on gastric carcinogenesis and progression is particularly important for early diagnosis and improving prognosis and survival of patients with advanced gastric cancer (Thrift and El-Serag, 2020).

The FNDC protein is characterized by at least one fibronectin type III domain (FN3) (Nie et al., 2020). They are necessary for various functions including tissue development and cell adhesion, migration and proliferation, and apoptosis. To date, FNDC5 is the most extensively studied FNDC, mainly because it is a launch vehicle for the peptide hormone irisin that was proposed to promote the conversion of white adipose tissue to beige adipose tissue (Waseem et al., 2022). Irisin has been shown to affect the proliferation of some cancer cells and the chemosensitivity of anticancer drugs like doxorubicin. Numerous studies have demonstrated that FNDC5 exerts differential effects on tumor cell proliferation and apoptosis in breast, lung, and liver cancer through multiple mechanisms (Kuloglu et al., 2016; Zhang et al., 2018; Nowinska et al., 2019; Pazgan-Simon et al., 2020; Cebulski et al., 2022; Taken et al., 2022). FNDC5 plays an important role in the occurrence, development, and metastasis of different tumors, suggesting that FNDC5 may serve as a potential target for tumor diagnosis and therapy (Pinkowska et al., 2021). Therefore, the expression and function of FNDC5 in tumors may be of great significance for the prevention and treatment of tumors.

The expression of FNDC5 and its value for the prognosis of gastric cancer patients were investigated. In addition, enrichment analysis was performed on genes co-expressed with FNDC5, and correlated regulatory genes were screened. A risk score and clinical prognosis prediction model for predicting OS in gastric cancer patients was constructed to evaluate the prognostic value of patients.

## Materials and methods

### Gene expression extraction

Clinical data of gastric cancer patients were collected from The Cancer Genome Atlas (TCGA) database (https://www.cancer.gov/tcga) and the Gene Expression Omnibus (GEO) database (https://www.ncbi.nlm.nih.gov/geo/). Five microarray gene expression datasets (GSE13195, GSE27342, GSE63069, GSE62254, and GSE65801) of gastric cancer patients were obtained from the GEO database. For the TCGA dataset, RNA sequencing data (FPKM values) were normalized into log2 (FPKM + 1). The method for extracting microarray gene expression values was based on our previous study.

### DNA constructs

pcDNA3.1-HA-FNDC5 (Jingkairui, Wuhan, China) was constructed to express full-length FNDC5 with an HA tag at the N-terminal end. pcDNA3.1-HA-FNDC5 was constructed by restriction enzyme sites BamH I and EcoR I site.

### Cell culture and transfection

SNU-1 and AGS cells were obtained from ATCC. both cells were cultured in RPM1 1640 medium (Biological Industries (BI), Israel) supplemented with 10% fetal bovine serum (FBS, BI), and cells were treated with 100 μg/ml penicillin and 100 μg/ml streptomycin (1% Pen/Strep) (BBI Life Sciences, Shanghai, China) at 37°C in a humid environment with 5% CO_2_. Cells were transfected using the VitalGENE-II *In Vitro* DNA Transfection Kit (biocanaan) according to the manufacturer’s instructions.

### Western blotting

Lysis of cells in Radioimmunoprecipitation Assay (RIPA) lysis buffer (Roche Ltd, Basel, Switzerland) containing protease inhibitors. Protein concentration in the lysates was measured by Micro BCA Protein Assay Kit (Pierce Biotechnology). Samples were separated on 10% SDS-PAGE and subsequently transferred to Amersham Protran nitrocellulose membranes (GE Healthcare Life Sciences, Fairfield, United States). The nitrocellulose membranes were then incubated with primary antibodies for the target proteins FNDC5 and GAPDH (Abmart) (Proteintech, Wuhan, China) at a dilution of 1:1,000 for 2 h. IRDye^®^ 800CW Goat Anti-Rabbit IgG or IRDye^®^ 680RD Goat anti-Mouse IgG (The proteins were detected and quantified using the Odyssey^®^ CLx Infrared Imaging System (LI-COR Biosciences).

### CCK8 assay

Cells were plated in 96-well plates at 2 × 10^6^ cells per well, for a total of 4 96-well plates. The first 96-well plate measured was set to 0 h, and then one 96-well plate was taken out every 24 h 10 μl of CCK8 solution was mixed with 100 μl of 1640 medium and added to each test sample before measurement, and the 96-well plate was incubated at 37°C and 5% CO_2_ for 1.5 h. Measure the OD of each well with a microplate reader, set to 450 nm, and collect and analyze the data. Four gradient times were measured: 0 h, 24 h, 48 h, and 72 h. The experiment was performed at least 3 times.

### Cell migration assay

When the cells were fully adhered to the cells and covered well (at least 95% confluence), a 10 μl pipette was used to gently scratch each well. After scratching, rinse the cells several times with PBS until any dead cells or debris floating in the culture wells are washed away. Replace the cultured cells with 2 ml of serum-free antibiotic-free medium. Six-well plates were photographed with an inverted phase contrast microscope at a time point we call “0 h”. The six-well plates were incubated at 5% CO_2_ and 37°C. After 48 h of incubation, the plates were rinsed with PBS and photographed again. Multiple views of each well were measured and three independent experiments were performed, which were repeated at least three times.

### Invasion assay

500 μl of complete medium was added to the 24-well plates, and Transwell chambers with matrix gel (Corning, New York, United States) were placed in the 24-well plates. The cell density was diluted to 1 × 10 ^4^ cells/mL by incubation in a 37°C incubator for 15 min 0.5 ml of cell dilution was added to each well of the Transwell chambers, and three replicate controls were added to each group. The medium was discarded and the cells in the inner basement membrane were removed with a cotton swab after 48 h (note that the lower outer layer of the basement membrane should not be touched to avoid wiping off the cells that had invaded). After fixation of the cells, approximately 300 μl of gentian violet dye solution was added to the Transwell for 10 min. After staining, the staining solution was discarded and excess dye was removed from the upper layer of the substrate membrane and the surrounding area with a cotton swab. The Transwell chamber was photographed with an inverted phase contrast microscope (Carl Zeiss).

### Immune infiltration analysis

The abundance of 22 types of immune cells was calculated by using CIBERSORT through sangerbox (http://sangerbox.com/Tool). And the correlation between FNDC5 expression and 22 types of immune cells in gastric cancer was also analyzed.

### Transcription factors identification

The Cistrome DB Toolkit database (http://dbtoolkit.cistrome.org) is a website that allows users to query TFs that might regulate the expression of genes. In the study, Cistrome DB Toolkit was performed to predict which TFs are most likely to decrease FNDC5 expression in gastric cancer.

### Methylation analysis

The methylation site and beta values of the FNDC5 promoter in gastric cancer were obtained from UCSC Xena Browser (http://xena.ucsc.edu/). At each CpG site, methylation is quantified by beta value to evaluate the degree of methylation.

### Weighted gene correlation network analysis

In the study, WGCNA was performed through sangerbox (http://sangerbox.com/Tool). The WGCNA hypotheses that the co-expression gene network follows the scale less distribution firstly, defines the adjoining function of the gene co-expression correlation matrix and gene network formation, calculates the different coefficients of different nodes, and constructs a hierarchical cluster tree accordingly finally.

### Functional enrichment analysis

To further analyze the potential functions of genes positively correlated with FNDC5 expression, the DAVID (http://david.abcc.ncifcrf.gov/) database was used. p < 0.05 was set as the cut-off criterion.

### Irisin

Irisin (Cat No.ADI-908-307-0010) was purchased from Enzo Life Sciences (Farmingdale, NY, United States).

### Statistical analysis

The statistical correlation was calculated using the *t*-test in this study. Overall survival (OS) and disease-free survival (DFS) were calculated by the Kaplan-Meier method, and differences between groups were tested by a log-rank test. All *p*-values < 0.05 were considered statistically significant.

## Results

### Expression and prognostic analysis of FNDC5 in patients with gastric cancer

**FIGURE 1:**
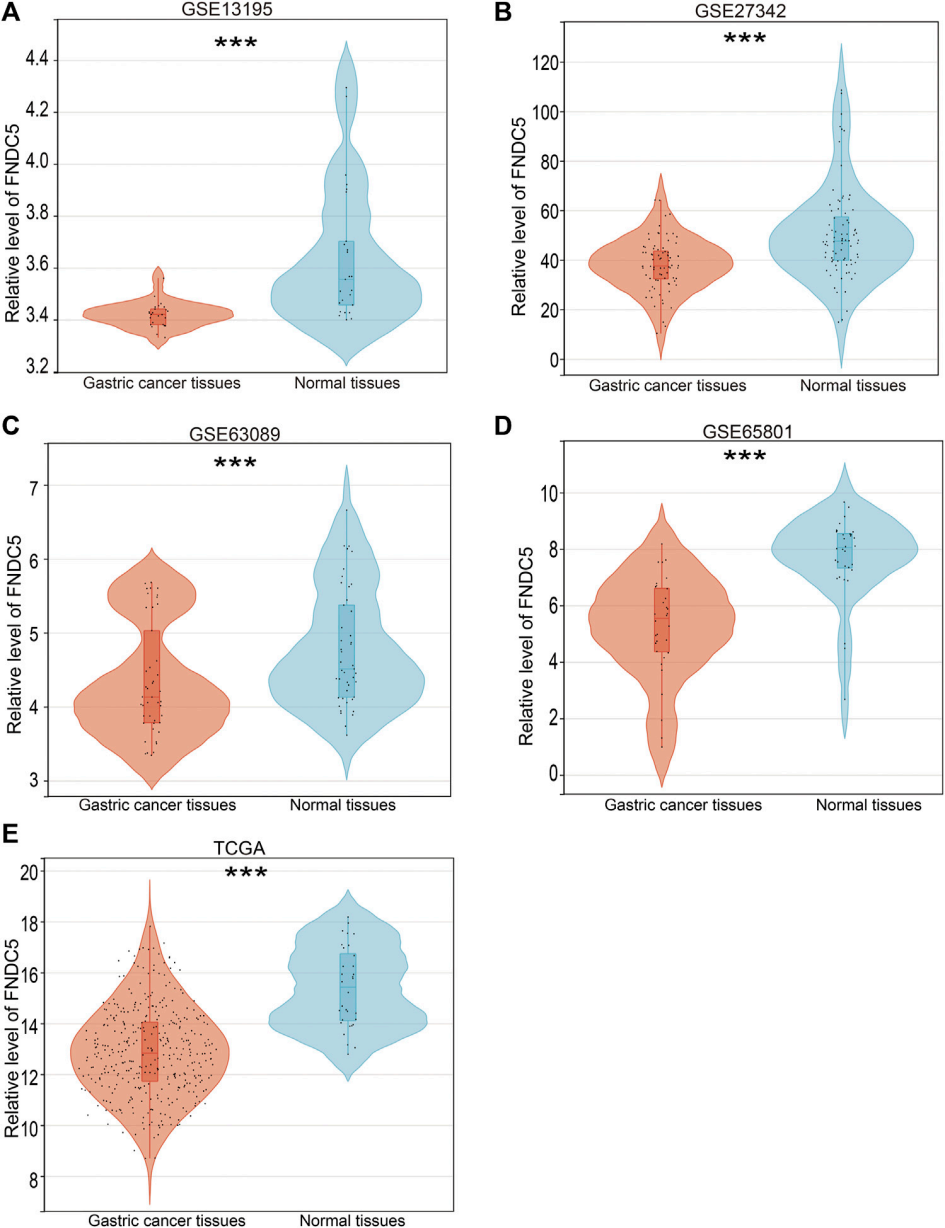
FNDC5 expression in gastric cancer based on GEO and TCGA databases. The mRNA levels of FNDC5 were analyzed in gastric cancer tissue and adjacent normal gastric tissue based on **(A)** GSE13195, **(B)** GSE27342, **(C)** GSE63069, **(D)** GSE65801, and **(E)** TCGA. ***, *p* < 0.001.

**FIGURE 2:**
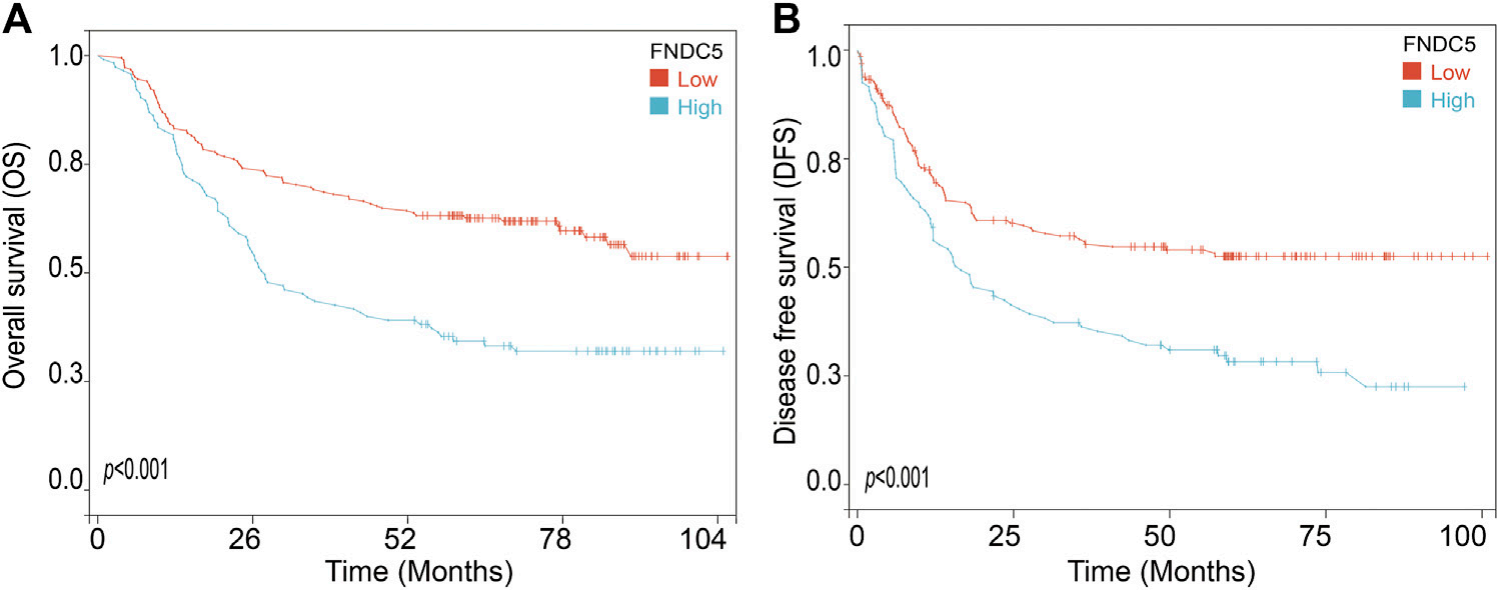
The effect of FNDC5 on the prognosis for gastric cancer patients. The **(A)** OS and **(B)** DFS analysis for FNDC5 in gastric cancer patients in GSE62254.

To investigate the FNDC5 expression model for gastric cancer, four related GEO datasets (GSE13195, GSE27342, GSE63069, and GSE65801) and TCGA datasets containing gastric cancer and adjacent normal gastric tissues were used. Analysis of these datasets showed that the expression of FNDC5 was decreased in gastric cancer tissues compared with adjacent normal gastric tissues (Figure 1; Supplementary Table S1). Gastric cancer is classified into two major histological subtypes, diffuse-type and intestinal-type adenocarcinoma. Based on TCGA dataset, we found that the expression of FNDC5 was decreased in intestinal-type adenocarcinoma compared with diffuse-type adenocarcinoma (Supplementary Figure S1). To identify whether the expression of FNDC5 is associated with overall survival (OS) and disease-free survival (DFS), 300 gastric cancer patients with both OS and DFS information in GSE62254 was analyzed. To our surprise, we observed that patients with low FNDC5 expression had significantly poorer OS and DFS (Figure 2; Supplementary Table S2).

### The relationship between the expression of FNDC5 and immune characteristics.

**FIGURE 3:**
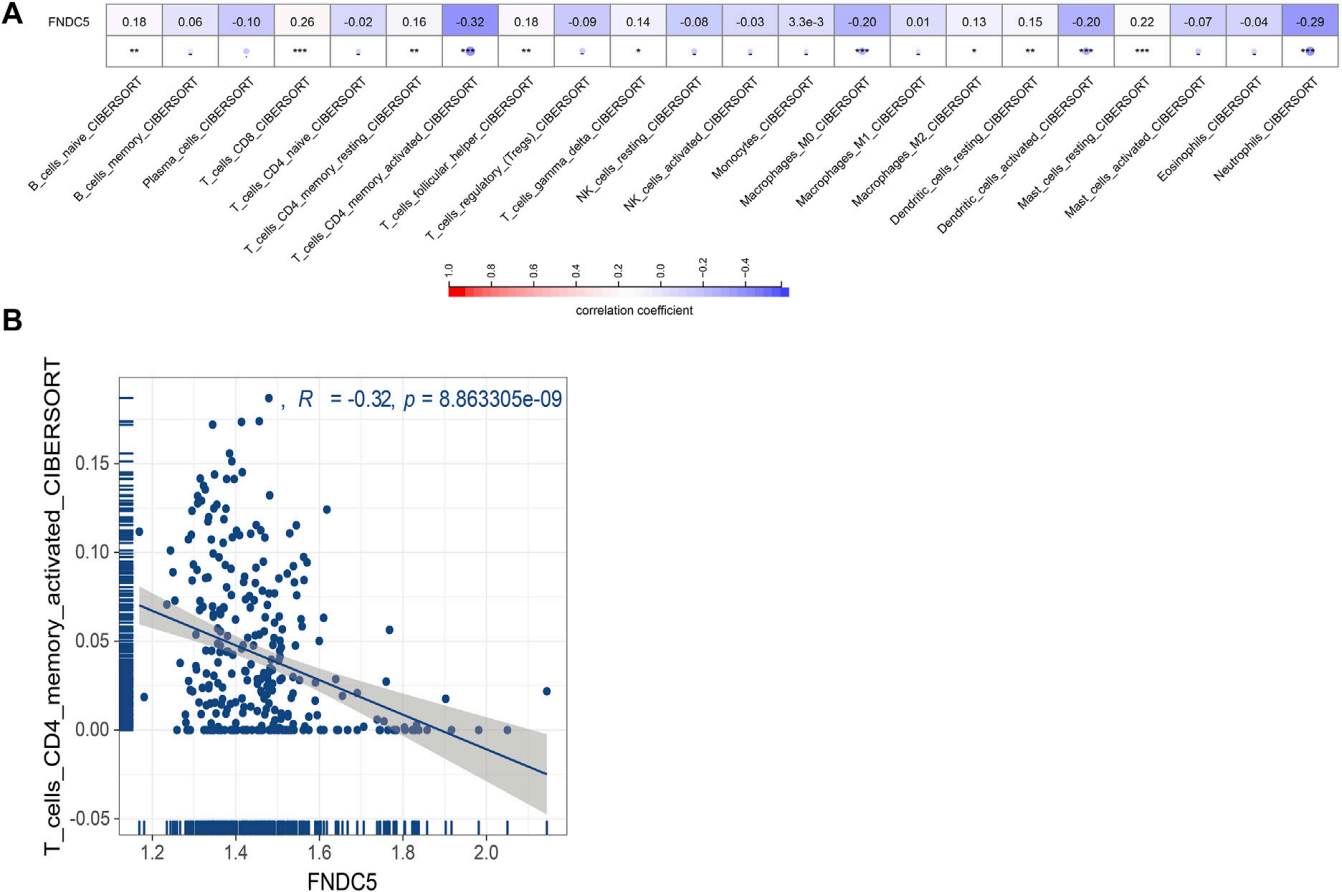
Correlations between FNDC5 and tumor immune microenvironment. **(A)** The association between FNDC5 and different immune cells. **(B)** Correlation between FNDC5 and T cells CD4 memory activated. *, *p* < 0.05; **, *p* < 0.01; ***, *p* < 0.001.

Next, we evaluated whether FNDC5 affected the tumor microenvironment and the effect on immune cells infiltrating inside the tumor. We estimated the correlation of FNDC5 expression with 22 immune cells based on signature expression data from gastric cancer patients and found a significant negative correlation between FNDC5 and CD4^+^ memory-activated cells by using the CIBERSORT algorithm (Figure 3; Supplementary Table S3).

### FNDC5/irisin inhibits the migration and invasion of gastric cancer cells

**FIGURE 4:**
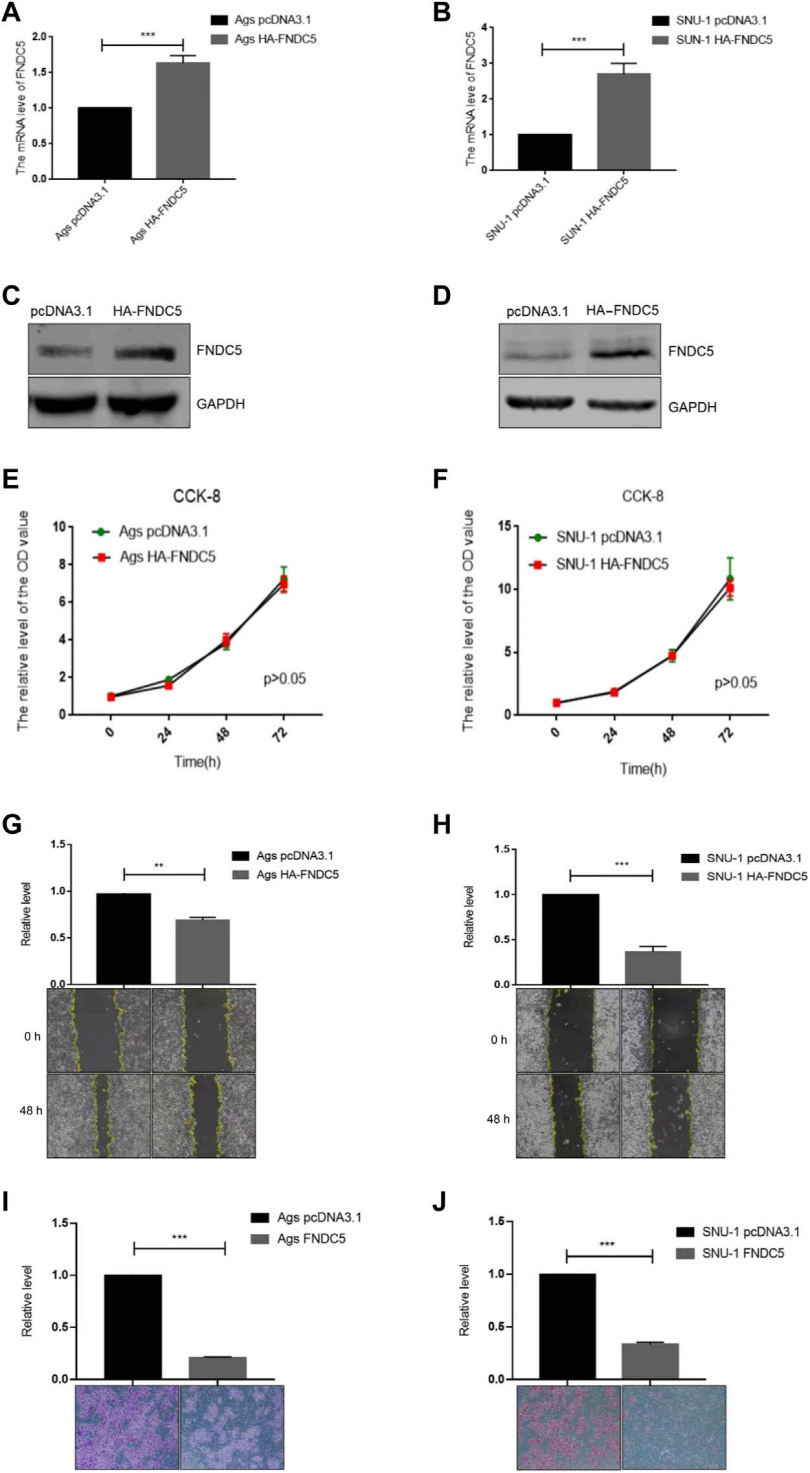
Overexpression of FNDC5 inhibited invasion and migration of gastric cancer cells. **(A,B)** mRNA levels of FNDC5 in Ags and SNU-1 cells were analyzed by overexpression of FNDC5 using RT-PCR. **(C,D)** Increased protein levels of FNDC5 in Ags and SNU-1 cells transfected with HA-FNDC5 using western blotting. **(E,F)** Using the CCK-8 assay to assess the impact of FNDC5 on cell growth. **(G,H)** Elevated expression of FNDC5 inhibits migration of Ags and SNU-1 cells. **(I,J)** Elevated expression of FNDC5 inhibits the invasion of Ags and SNU-1 cells. **, *p* < 0.01; ***, *p* < 0.001.

**FIGURE 5:**
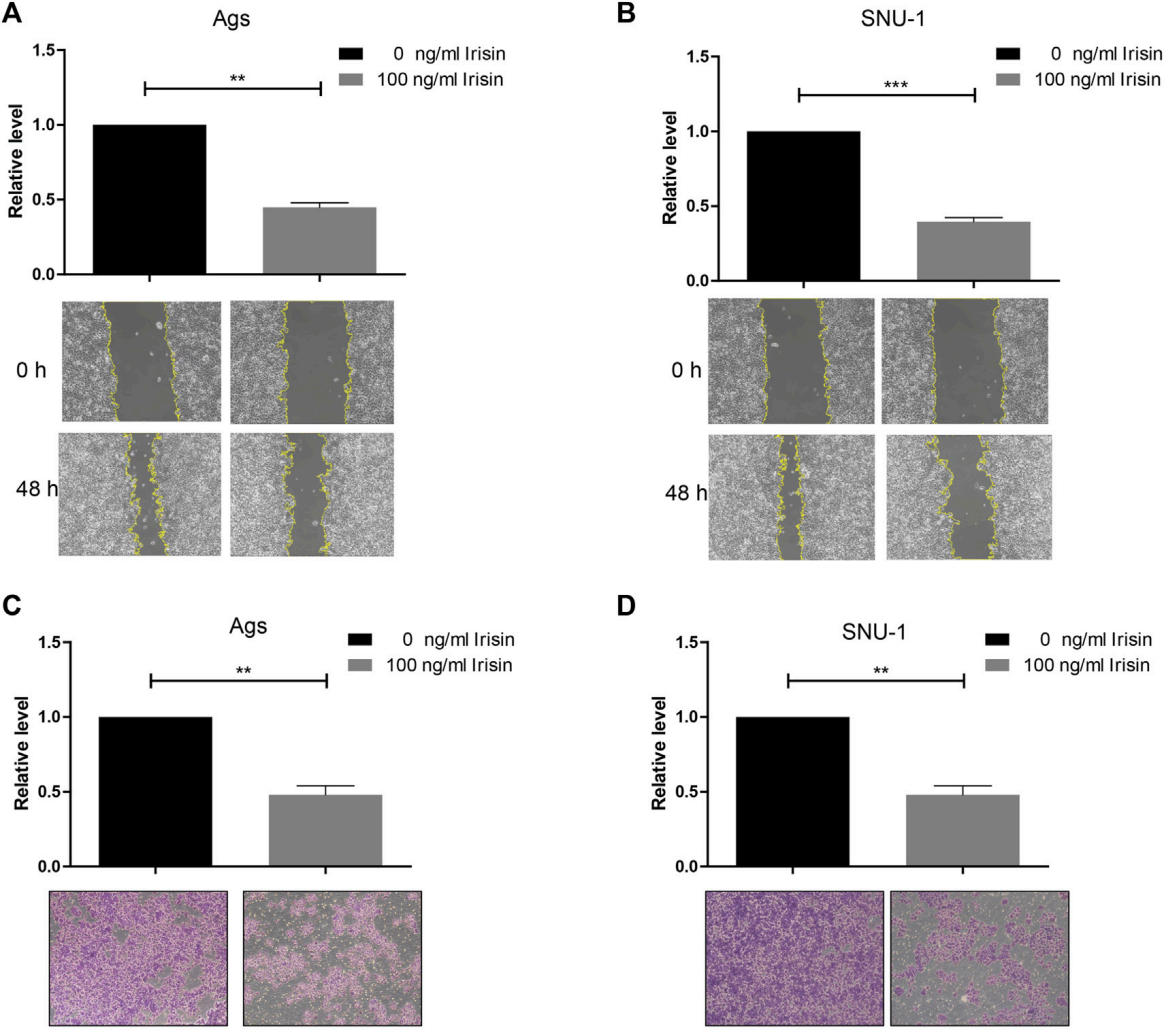
Irisin inhibited invasion and migration of gastric cancer cells. **(A,B)** Irisin inhibits migration of **(A)** Ags and **(B)** SNU-1 cells. **(C,D)** Irisin inhibits the invasion of **(C)** Ags and **(D)** SNU-1 cells. **, *p* < 0.01; ***, *p* < 0.001.

To investigate the impact of FNDC5 on gastric cancer cell proliferation, migration, and invasion, we transfected SNU-1 and AGS cells with HA-FNDC5. Then, we examined FNDC5 expression using RT-PCR and western blot. FNDC5 mRNA and protein were markedly expressed in SNU-1 and AGS cells as reflected by RT-PCR (Figures 4A,B) and western blot analysis (Figures 4C,D). The functional experiments showed that FNDC5 overexpression did not affect the proliferation of SNU-1 and AGS cells (Figures 4E,F), but inhibited the migration (Figures 4G,H) and invasion (Figures 4I,J) of SNU-1 and AGS cells. In addition, we explored the role of irisin in gastric cancer by the addition of exogenous irisin. The results suggest that irisin can suppress the migration and invasion of gastric cancer cells (Figure 5).

### Identification of regulatory transcription factors and methylation sites of FNDC5

**FIGURE 6:**
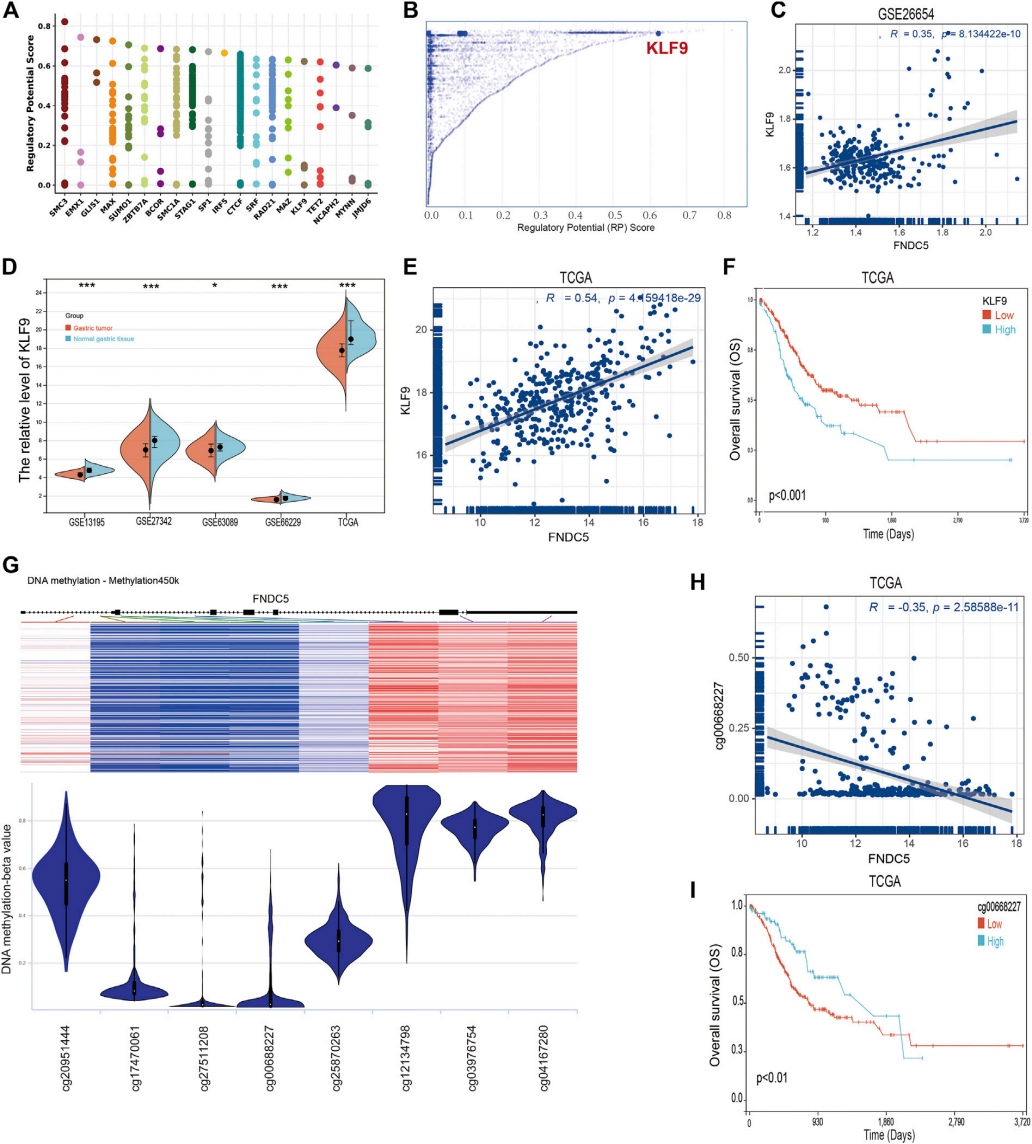
TFs and DNA methylation sites can regulate the expression of FNDC5. **(A)** Top 20 TFs likely to regulate FNDC5 in different human cancers. **(B)** TFs with high regulatory potential (10 k distance from TSS) **(C)** Correlation between FNDC5 and KLF9 mRNA expression in gse62254 database. **(D)** KLF9 expression in gastric cancer in five mRNA datasets. **(E)** Correlation analysis of FNDC5 and KLF9 in gastric cancer patients. **(F)** OS analysis of KLF9 for gastric cancer patients in TCGA database. **(G)** Association of FNDC5 with DNA methylation in gastric cancer. **(H)** Association of FNDC5 with methylation site cg00668227. **(I)** OS analysis of methylation sites (cg00668227) for gastric cancer patients in the TCGA database. *, *p* < 0.05; ***, *p* < 0.001.

To identify members of a putative molecular network that regulates the expression of FNDC5, we investigated transcription factors (TFs) that can affect the transcription of the FNDC5 gene. First, the top 20 regulatory transcription factor TFs in human cancers were identified using the Cistrome DB toolkit (Figure 6A), and KLF9 was found to have the greatest regulatory potential (Figure 6B). Additionally, KLF9 expression is significantly positively correlated with FNDC5 in the GSE62254 database (Figure 6C). Next, the expression of KLF9 in gastric cancer tissues and adjacent tissues was analyzed by GEO dataset (GSE13195, GSE27342, GSE63069, and GSE65801) and TCGA database (Figure 6D). The results of the analyses revealed that KLF9 expression was also decreased in gastric cancer tissues compared with normal gastric tissues. A significant positive correlation was also observed between KLF9 expression and FNDC5 expression in TCGA database (Figure 6E). We also found that the low expression of KLF9 had a better prognostic value (Figure 6F), which was the same as the expression and prognostic analysis of FNDC5. Therefore, KLF9 may play a vital role in regulating the expression of FNDC5 in gastric cancer.

Methylation is one of the major mechanisms regulating gene expression and has been demonstrated to control transcription. Studies have demonstrated that increased expression of multiple genes is associated with promoter hypomethylation. The association between FNDC5 and promoter methylation in gastric cancer patients was detected by UCSC Xena. Figure 6G shows the chip quantification of the association between methylation of eight FNDC5 CpG sites and FNDC5 expression (Supplementary Table S4). Only the Pearson correlation coefficient between the methylation site (cg00668227) and FNDC5 was −0.35 (*p* < 0.05), which suggested that the methylation site (cg00668227) had an adverse significant correlation with FNDC5 expression (Figure 6H). Prognostic analysis showed that the overall survival time of gastric cancer patients was prolonged when a methylation site (cg00668227) was more highly methylated (Figure 6I; Supplementary Table S5). Generally, these results suggest that TFs and DNA methylation modifications potential have an important effect on the occurrence and development of gastric cancer by regulating FNDC5 expression.

### Enrichment analysis of FNDC5 co-expressed genes in gastric cancer

**FIGURE 7:**
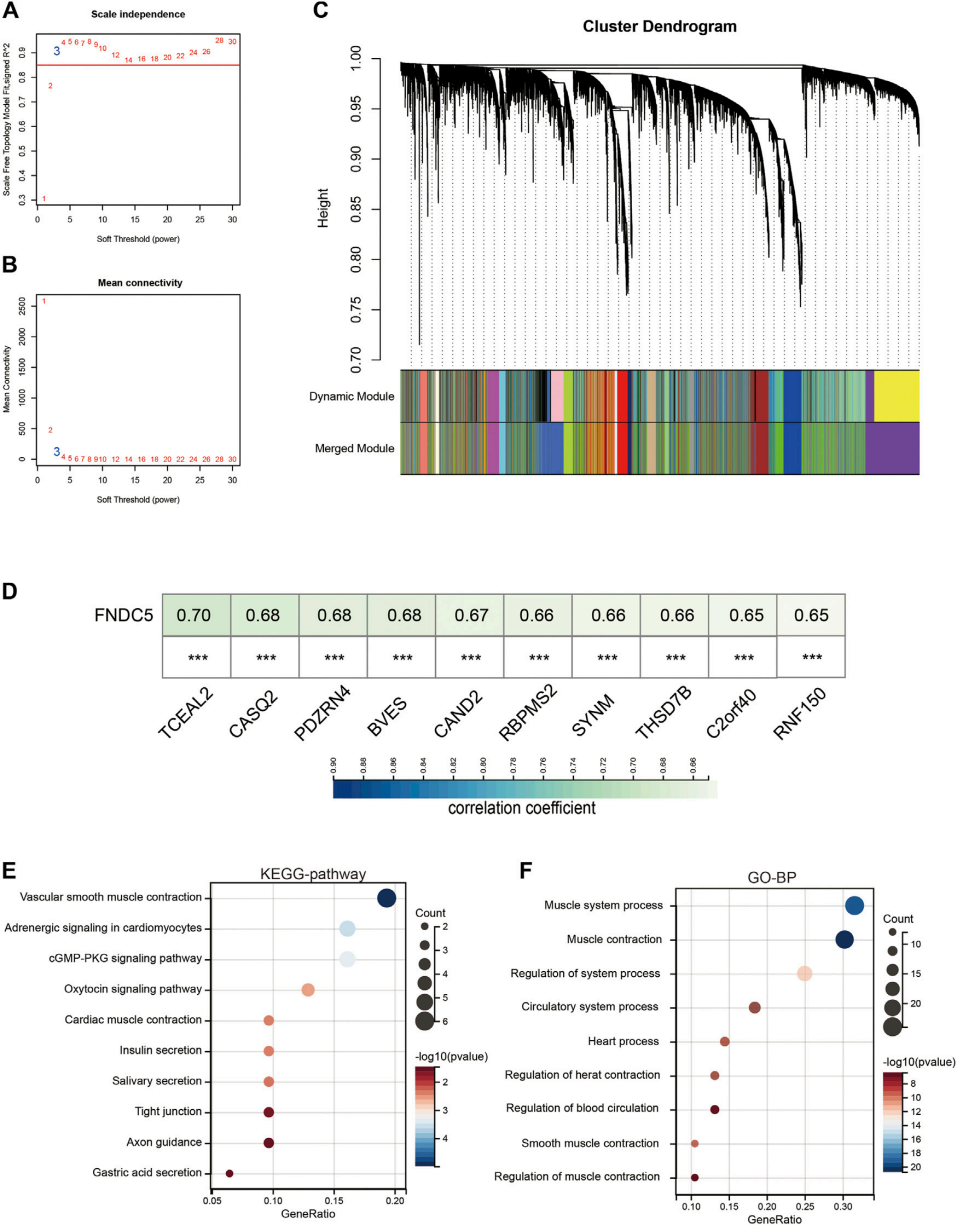
Co-expression module genes associated with FNDC5 were identified using WGCNA. **(A)** Relationship between scale-free topological model fit and soft threshold (power) in the GSE62254 database. **(B)** Relationship between average connectivity and various soft thresholds in the GSE62254 database. **(C)** Dendrogram of modules identified by WGCNA. **(D)** The top ten genes most associated with FNDC5 expression in GSE62254. **(E,F)** The **(E)** KEGG pathway and **(F)** GO-BP enrichment analysis of these FNDC5-related 1156 genes. ***, *p* < 0.001.

To reveal the highly correlated gene and co-expression network of the FNDC5 gene in gastric cancer patients, WGCNA analysis was performed. Gastric cancer samples were obtained from the GSE62254 database for constructing the WGCNA network. We calculated the network topology for soft threshold power from 1 to 30 to choose the best threshold. One of the most critical parameters in the construction of WGCNA networks is the power value, which affects the average connectivity and independence of each co-expression module. A power value of 3 is the minimum power for a scale-free topology (Figures 7A,B). The co-expression similarity matrix was transformed into an adjacency matrix, and the topological overlap matrix (TOM) was calculated. The dynamic tree-cut analysis produced different colored modules, among which 2657 genes co-expressed with FNDC5 belonged to blue modules (Figure 7C; Supplementary Table S6). The results of correlation analysis showed that 1156 genes out of 2567 genes were positively correlated with FNDC5 expression. The top ten genes, including TCEAL2, CASQ2, PDZRN4, BVES, CAND2, RBPMS2, SYNM, THSD7B, C2orf40, and RNF150, most associated with FNDC5 expression are shown in Figure 7D (Supplementary Table S7). Meanwhile, to further characterize the functions of the 1156 genes, we analyzed the GO and KEGG pathways respectively through the DAVID database. KEGG pathway enrichment network analysis showing FNDC5-related 1156 genes enriched in vascular smooth muscle contraction, adrenergic signaling in cardiomyocytes, cGMP-PKG signaling pathway, oxytocin signaling pathway, cardiac muscle contraction, insulin secretion, salivary secretion, tight junction, axon guidance, gastric acid secretion. (Figure 7E; Supplementary Table S8). GO-BP enrichment network analysis showing FNDC5-related 1156 genes enriched in muscle system process, muscle contraction, regulation of system process, circulatory system process, heart process, regulation of heart contraction, regulation of blood circulation, smooth muscle contraction, regulation of muscle contraction (Figure 7F; Supplementary Table S9).

### Prognosis model of FNDC5-related genes constructed and validated for gastric cancer patients

**FIGURE 8:**
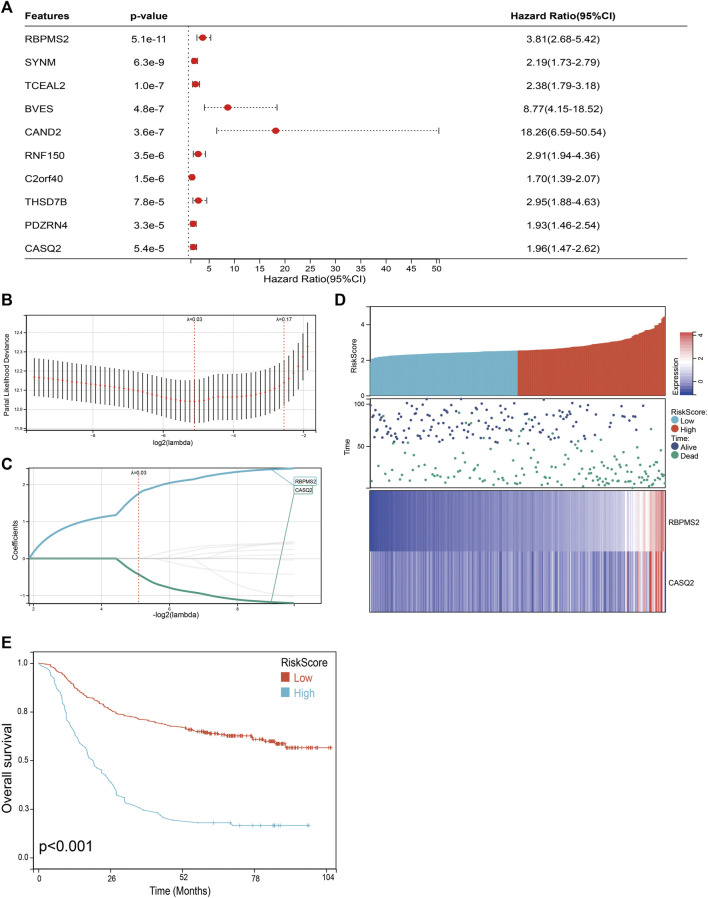
Construction of a classifier to predict OS for gastric cancer patients. **(A)** Univariate Cox regression analysis was used to assess the genes that related to prognosis. **(B)** Partial likelihood deviation of the OS of the LASSO coefficient profile. **(C)** LASSO coefficient curves of RBPMS2 and CASQ2 expressions. **(D)** The distribution of risk score, survival status, and mRNA expression levels of gastric cancer patients in GSE62254 database. **(E)** Kaplan-Meier curves to compare overall survival of low-risk and high-risk groups.

We next performed a univariate Cox survival analysis on the top 10 genes most associated with FNDC5 expression. And the results indicated that these 10 FNDC5-related genes were associated with the prognosis of gastric cancer patients. The high level of these 10 FNDC5-related genes was significantly correlated with shorter OS for gastric cancer patients (Figure 8A). Then these 10 FNDC5-related genes were used to construct a prognostic model using Lasso-Cox proportional hazards regression, and the resulting best prognostic signature for predicting overall survival consisted of two FNDC5 co-express genes including RBPMS2 and CASQ2. Risk Score = (1.759 * RBPMS2 expression) + (−0.433 * CASQ2 expression) (Figures 8B,C). A risk score was assigned to each gastric cancer patient according to a risk scoring formula and divided into a low-risk score group and a high-risk score group. The distribution of risk scores, survival status, and mRNA expression levels of gastric cancer patients in the GSE62254 database is shown (Figure 8D). Kaplan-Meier curve analysis showed that the overall survival of the low-risk group was distinctly longer than that of the high-risk group (Figure 8E; Supplementary Table S10).

**FIGURE 9:**
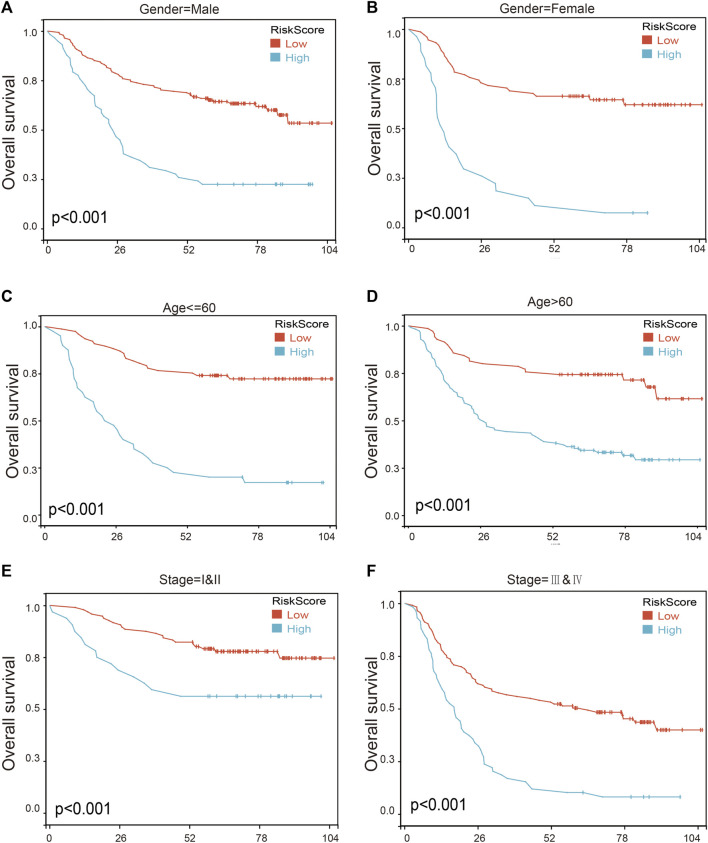
The survival analysis in different subgroup of gastric cancer patients according to risk score model. **(A,B)** Prognostic analysis of gastric cancer patients in gender = female **(A)** and gender = male **(B)** subgroups. Prognostic analysis of gastric cancer patients in **(C,D)** Age ≤ 60 **(C)** and age >60 **(D)** subgroups. **(E,F)** Prognostic analysis of gastric cancer patients in subgroups of stage = I and II **(E)** and stage = III and IV **(F)** subgroups.

To further assess the robustness of the risk score model, we stratified the gastric cancer patients based on gender, age, and tumor stage in GSE62254 database. Survival analyses indicated that gastric cancer patients with high-risk scores were found to have worse outcomes in different stratification (Figure 9; Supplementary Table S11). These results further confirmed the relatively good stratification ability of the prognostic model.

**FIGURE 10:**
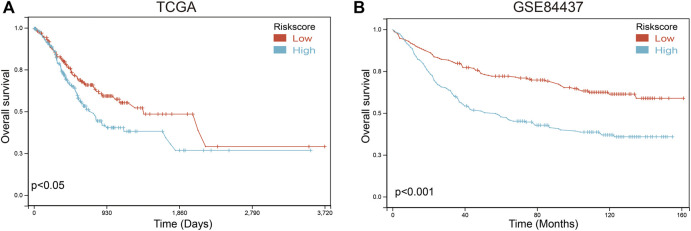
Validation of the prognosis risk model The prognosis risk model was validated using TCGA **(A)** and GSE84437 **(B)** databases.

To further verify the validity and stability of the prognostic model, TCGA and GSE84437 databases were used. Each patient was brought into the previous prognostic model to calculate the risk score. Patients were divided into high-risk and low-risk groups. Kaplan-Meier curve analysis showed that gastric cancer patients with low-risk scores had a better OS than those in the high-risk-score group in TCGA (Figure 10A; Supplementary Table S12) and GSE84437 databases (Figure 10B; Supplementary Table S13), indicating good accuracy.

### The construction of a clinical prognosis prediction model

**FIGURE 11:**
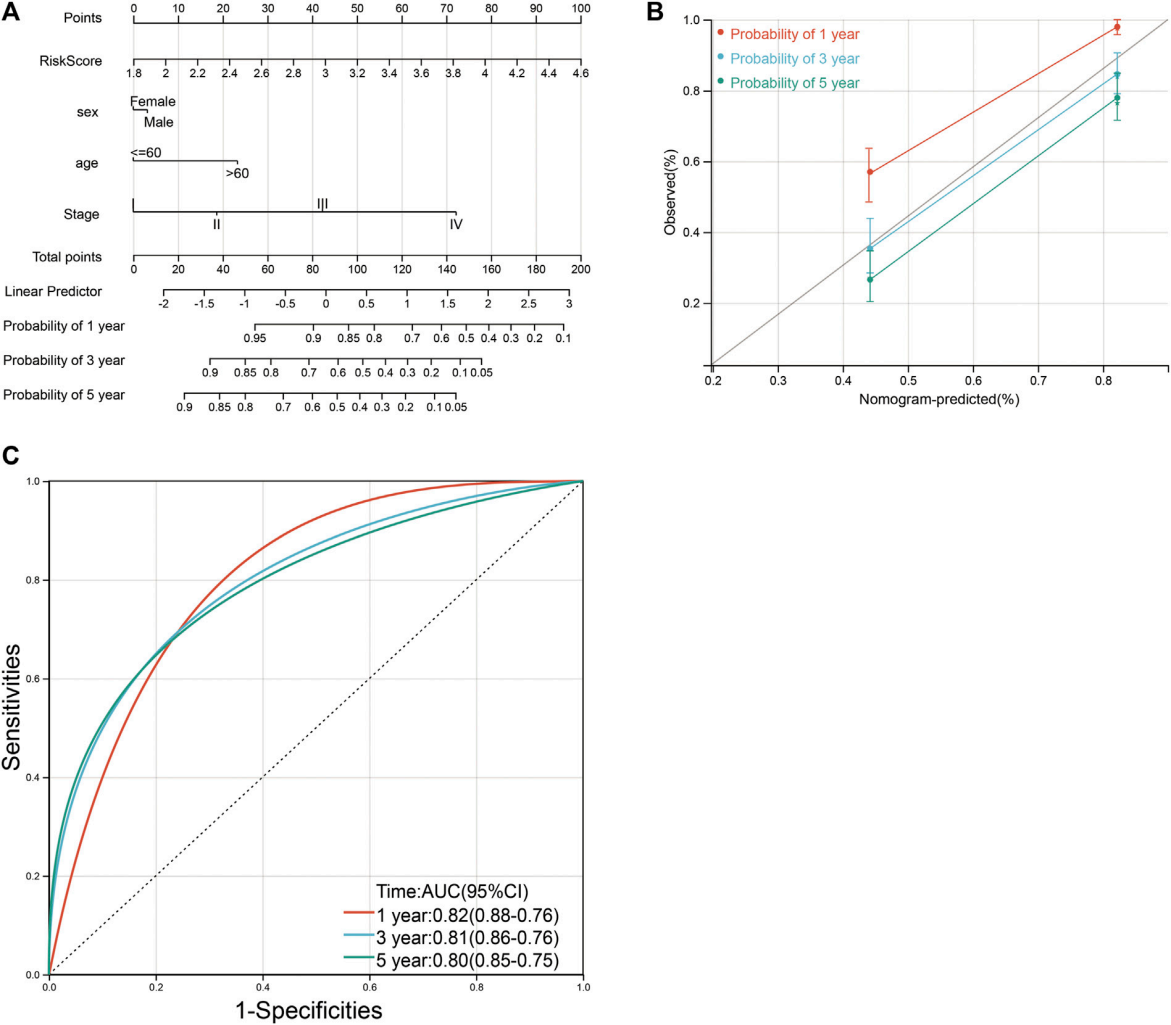
Nomograms and calibration plots for predicting outcomes in gastric cancer patients based on risk scores. **(A)** Nomogram for predicting 1-, 3-, and 5-year events, combining clinical data with age, sex, tumor grade, and risk score. The line segment corresponding to each variable is marked with a scale, indicating the value range of the variable, and the length of the line segment reflects the contribution of the factor to the resulting event. The Point in the figure represents the individual score corresponding to each variable under different values, and the sum of the corresponding individual scores after taking all the variables. **(B)** Calibration plot for predicting overall survival. **(C)** ROC curve was used to verify the diagnosis with AUC at 1-, 3-, and 5-years.

Based on four variables including age, sex, tumor grade, and risk score, survival nomograms were created to accurately calculate 1-, 3-, and 5-year survival probabilities (Figure 11A; Supplementary Table S11). The overall C-index of the model is 0.75, which showed excellent calibration of the nomogram (Figure 11B). ROC curve was used to verify the diagnostic effect and AUC was found to be greater than 0.8 regardless of 1-year (AUC, 0.82; 95% CI, 0.76–0.88), 3-year (AUC, 0.81; 95% CI, 0.76–0.86) and 5-year (AUC, 0.80; 95% CI, 0.75–0.85) (Figure 10C), suggesting that this nomogram was reliable and robust. We believe that nomograms may have good accuracy for survival prediction in gastric cancer patients.

## Discussion

FNDC5 consists of 209 amino acids and can be divided into four domains including signal peptide (SP), fibronectin III domain (FND), hydrophobic domain (H), and C-terminal domain (C) (Waseem et al., 2022). FNDC5 is proteolytically hydrolyzed at amino acid position 30 and position 142 under the action of peroxisome proliferator-activated receptor gamma coactivator 1-α (PGC1-α) to produce irisin. FNDC5 protein range in mass from 20 to 32 kDa, and this difference is related to post-translational modifications (Boström et al., 2012). Numerous studies have shown that FNDC5 plays an important role in cancer diseases, including many types of malignancies, namely breast cancer, lung cancer, reproductive tract cancer, and bone cancer (Zhang et al., 2018; Cheng et al., 2020; Kim et al., 2021; Pinkowska et al., 2021; Cebulski et al., 2022; Zhu et al., 2022). In lung cancer, FNDC5 inhibits EMT and reduces the migration and invasion abilities of lung cancer cells by mediating the PI3K/AKT/Snail signaling pathway (Shao et al., 2017). FNDC5 was shown to inhibit EMT through the modulation of STAT3/Snail pathway in osteosarcoma (Kong et al., 2017). In pancreatic cancer, FNDC5 inhibited the growth of pancreatic cancer cells through the AMPK-mTOR pathway, and inhibited the migration and invasion of pancreatic cancer cells through the inhibition of EMT (Liu et al., 2018). Moreover, Zhang Z. et al. found that serum irisin is decreased in breast cancer patients with spinal metastasis compared to non-metastatic patients (Zhang et al., 2018).

FNDC5/Irisin has been shown to aid the diagnosis for several cancers. Increased levels of FNDC5 are associated with a reduced risk of breast cancer, and FNDC5 can be used for differential diagnosis and prognosis of breast cancer (Zhang et al., 2018).The serum irisin level in renal cancer patients was significantly higher than that in healthy controls suggesting that serum irisin could be used as a biomarker for the diagnosis of renal cancer (Thomas et al., 2017). Serum irisin protein was increased in gastric cancer and increased FNDC5 expression may have a cachexia effect in cancer-induced mice (Us Altay et al., 2016). Serum irisin level was significantly lower in the bladder cancer patients compared to the control group, demonstrating that serum irisin levels can be used for the diagnosis of bladder cancer (Taken et al., 2022). The level of serum irisin in prostate cancer patients is considerably reduced and irisin may be used as a biomarker for prostate cancer patients (Aslan et al., 2020). There are many advantages for irisin in serum to be promising biomarkers. First, Serum samples are more easily accessible compared with tumor tissue samples. Second, sufficient irisin in serum makes it easy to be determined by simple and fast methods.

Gastric cancer is the second leading cause of cancer death worldwide, mainly due to the poor prognosis, with an average 5-year survival rate of less than 20% and asymptomatic onset in the early stage (Chia and Tan, 2016). The expression analysis from gastric cancer-related GEO databases (GSE13195, GSE27342, GSE63089, and GSE65801) and TCGA database showed that the expression of FNDC5 was significantly downregulated in gastric cancer tissues compared with matched adjacent normal gastric tissues. Based on TCGA dataset, we found that the expression of FNDC5 was decreased in intestinal-type adenocarcinoma compared with diffuse-type adenocarcinoma. However, it confused us when Kaplan-Meier analysis showed that gastric cancer patients with high expression of FNDC5 had shorter overall survival than patients with low levels.

Then, we investigated the impact of FNDC5 in proliferation, migration and invasiveness of gastric cancer cells. These results indicated that overexpressed FNDC5 had no effect on the proliferation of gastric cancer cells but inhibited the migration and invasion of gastric cancer cells *in vitro*. Numerous studies have shown its impact on the proliferation of cancer cells (Waseem et al., 2022). It was demonstrated that irisin can activate the AMPK pathway and downregulates the mTOR pathway, thereby suppressing pancreatic cancer cell growth (Liu et al., 2018). The number of malignant breast tumor cells decreased significantly upon exposure to irisin (Gannon et al., 2015). The increased level of irisin leads to decreased proliferation in a lung cancer cell by inhibiting the PI3K/Akt pathway (Shao et al., 2017). These studies suggested that FNDC5 may have different functions in different tumor cells.

Tumor progression is a process of continuous proliferation, infiltration, and migration of cancer cells in the complex tumor microenvironment, which contains a variety of different immune cells. Changes in the status of tumor-reactive immune cells are associated with clinical changes in patient survival as well as immune tolerance response (Moreau et al., 2022). We investigated the association of FNDC5 with various immune cells in the tumor microenvironment and found a significant negative correlation between FNDC5 and CD4^+^ memory T cells, implying an increase in the number of CD4^+^ memory T cells when FNDC5 was lowly expressed. After induction of the immune response, antigen-reactive T cell persist in the memory pool and provide systemic immune surveillance in lymphoid organs. Increasing CD4^+^ memory cell density in gastric cancer has been reported to be a predictor of prolonged patient survival, while further studies have shown that CD4^+^ T cells are more infiltrated in gastrointestinal tumors compared to normal tissue (Ge et al., 2019). The results of immune cell clustering and clinicopathological characterization show that high levels of activated CD4^+^ memory T cells are significantly associated with a better prognosis (Ning et al., 2020).

Therefore, the better prognosis of low FNDC5 expression can be attributed to the increased number of CD4^+^ memory activated T-cell infiltration in tumors, but the exact mechanism of the effect needs to be further explored.

Meanwhile, we identify the regulatory transcription factors and methylation sites of FNDC5. We used multiple databases to carry out enrichment analysis of FNDC5-expressed genes and found that the expression level of KLF9 in gastric cancer patients was favorably correlated with FNDC5. The TCGA database was referenced simultaneously to determine whether FNDC5 levels were manipulated by modulating DNA methylation in gastric cancer. The results showed that FNDC5 expression was significantly negatively correlated with the level of methylation site (cg00668227). These results suggest that the low expression of FNDC5 in gastric cancer may be related to the expression of transcription factor KLF9 and the methylation of cg00668227 locus.

Next, we analyzed the genes highly associated with FNDC5 in gastric cancer patients by WGCNA, and found that FNDC5-related genes were enriched in muscle system process, muscle contraction, and so on. Considering irisin, an adipocytokine secreted by FNDC5, is a hormone-like myokine produced in abundance by skeletal muscle in response to exercise, both in mice and humans (Us Altay et al., 2016).

Using Cox univariate analysis and Lasso regression, we constructed a prognostic risk model for gastric cancer patients including 2 FNDC5-related genes. Meanwhile, we conducted external validation, and subgroup analysis to assess the reliability of the risk prognostic model. The AUC values of the ROC curves of 1-, 3-, and 5-year survival of the model were all greater than 0.8, which indicated that the signature composed of 2 FNDC5-related genes had good performance in predicting the prognosis of gastric cancer patients.

There are some limitations in the current study which need to be acknowledged. First, there is a contradiction between *in vitro* results and clinical data. The change of tumor microenvironment could be one of the reasons for the contradiction between *in vitro* results and clinical data. However, it does not indicate immune cells are the major cause of the contradictory clinical results. There are many other possibilities besides it, for example, the efficiency of the T cells, the differentiation of immune cells, or even gastric microbial community. To answer the question, conditional knock mice will be required. For example, using APC mice that have specific knockout of FNDC5 in T lymphocytes or gastric epithelial cells. Second, the hypothesis that KLF9 might be the transcription factor of FNDC5 should been further confirm by immunofluorescence, chromatin immunoprecipitation and dual-luciferase assay.

## Conclusion

Overall, we found that low levels of FNDC5 companying increasing CD4^+^ memory activated cells were a good prognostic factor in patients. Our experimental results suggest that FNDC5 inhibits the invasion and migration of gastric cancer cells. Meanwhile, we found that FNDC5 expression correlates with DNA methylation and the TF gene KLF9. Furthermore, the risk score model including FNDC5-related genes can be used to predict the prognosis of gastric cancer patients, leading to improved monitoring of the present patient population.

## Data Availability

The datasets presented in this study can be found in online repositories. The names of the repository/repositories and accession number(s) can be found in the article/Supplementary Material.
