# Supplementary figures and images for: Low FNDC5/Irisin expression is associated with aggressive phenotypes in gastric cancer
Source: Front Pharmacol. 2022 Oct 28;13:981201. doi: 10.3389/fphar.2022.981201 (PMC9649517; doi:10.3389/fphar.2022.981201)

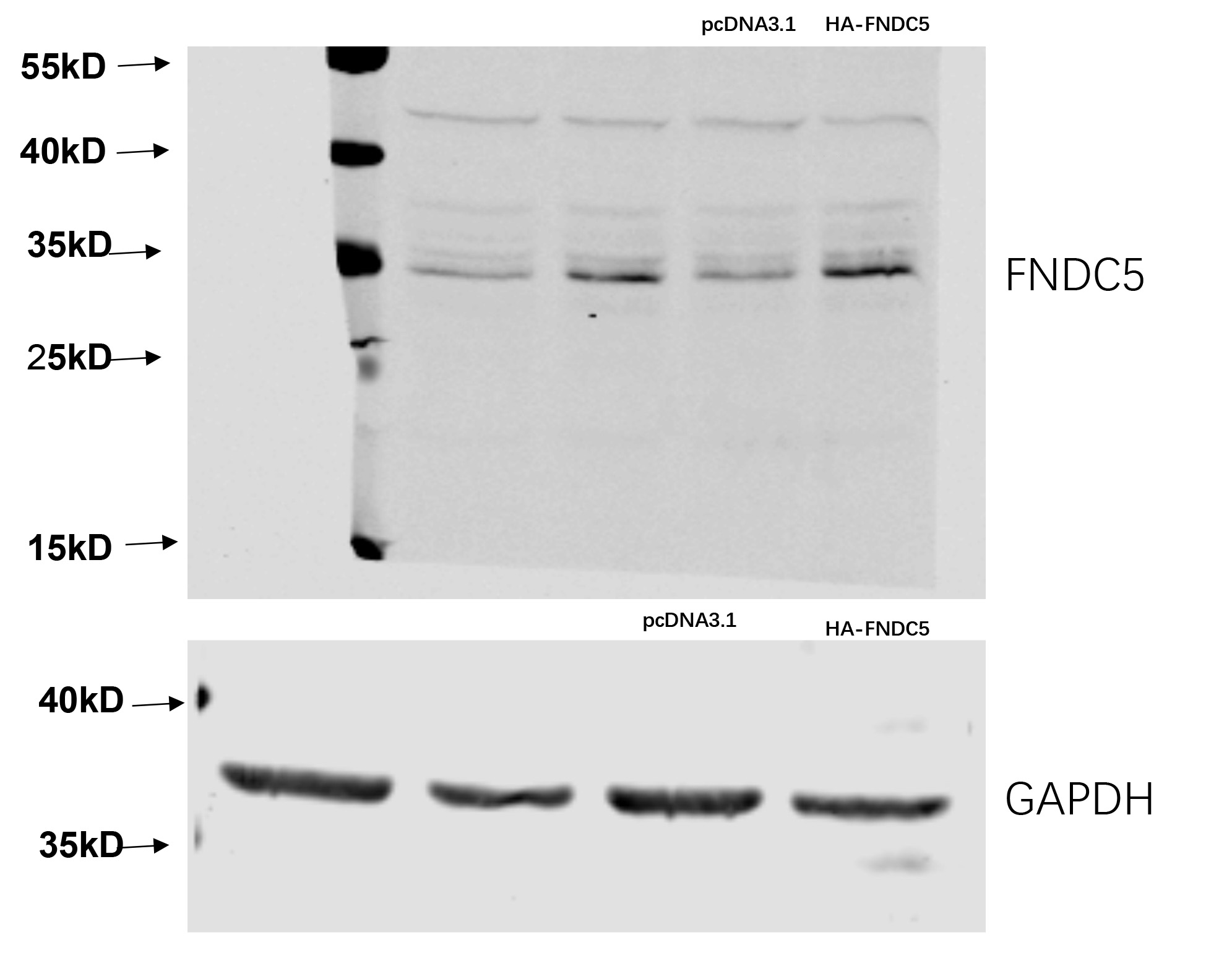

Supplement: Supplementary file 2 [file Image3.JPEG]

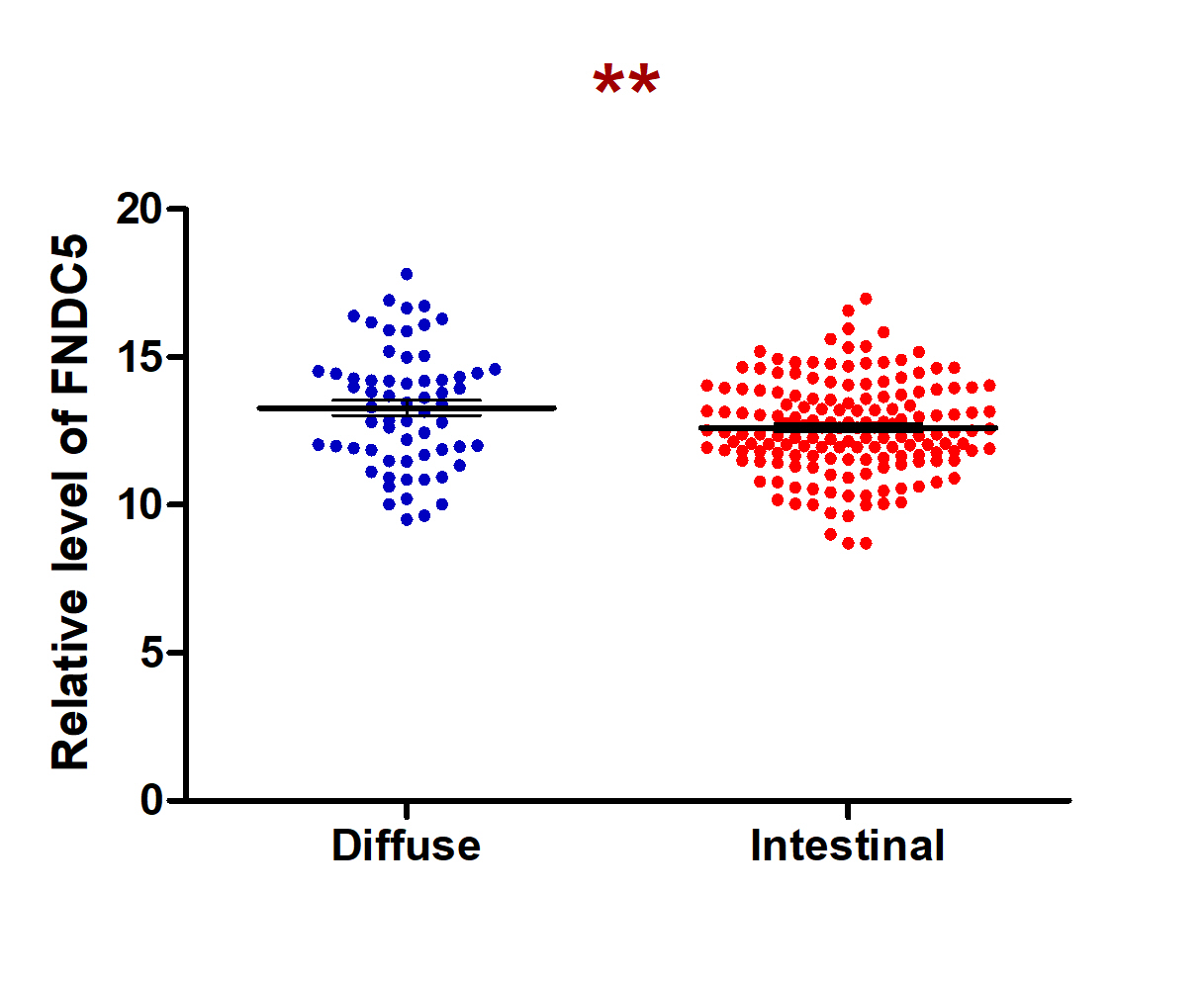

Supplement: Supplementary file 5 [file Image1.JPEG]

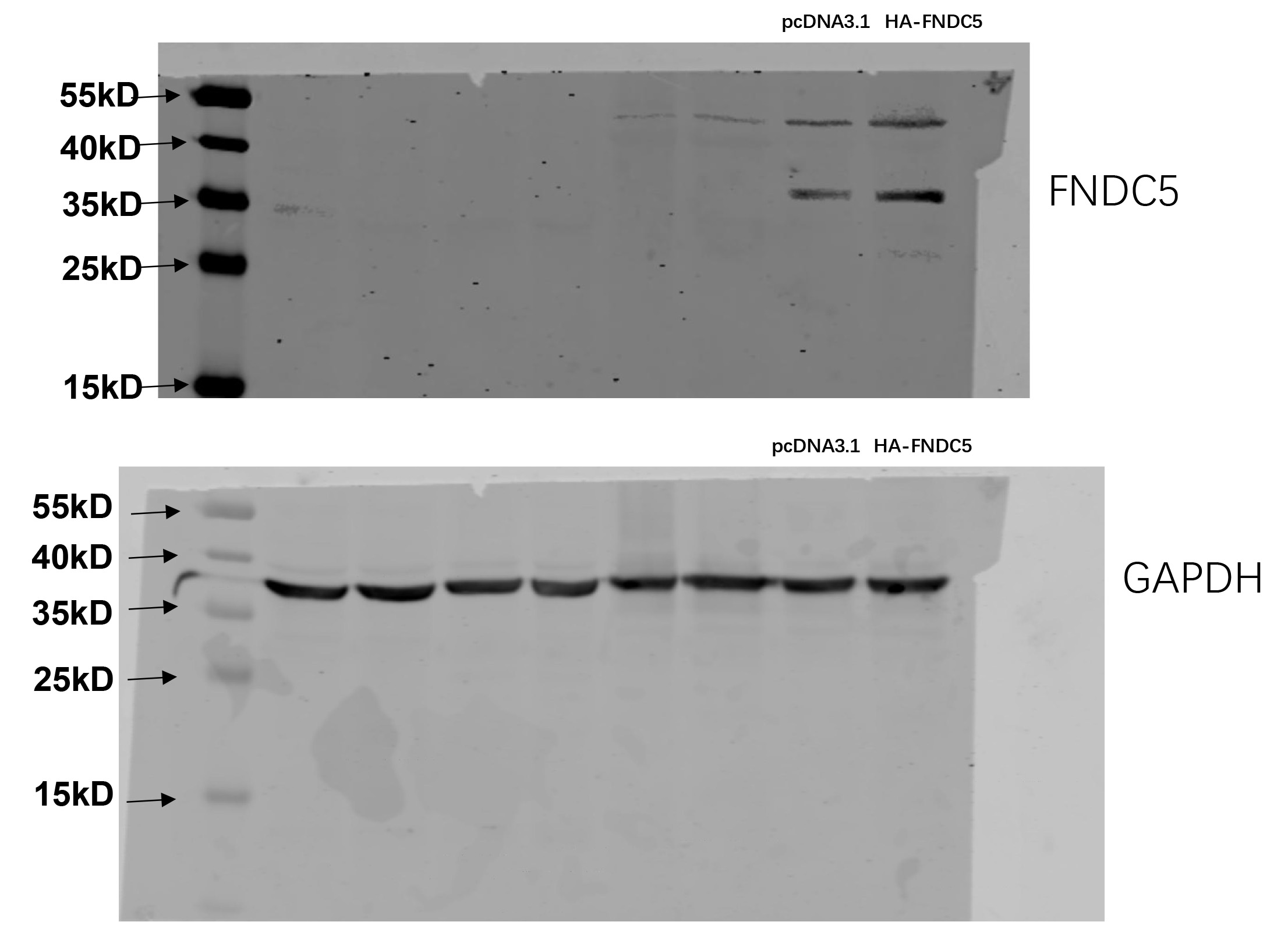

Supplement: Supplementary file 6 [file Image2.JPEG]
